# Vascular Permeability Drives Susceptibility to Influenza Infection in a Murine Model of Sickle Cell Disease

**DOI:** 10.1038/srep43308

**Published:** 2017-03-03

**Authors:** Erik A. Karlsson, Thomas H. Oguin, Victoria Meliopoulos, Amy Iverson, Alexandria Broadnax, Sun-Woo Yoon, Tamara Pestina, Paul Thomas, Richard Webby, Stacey Schultz-Cherry, Jason W. Rosch

**Affiliations:** 1 Department of Infectious Diseases, St. Jude Children’s Research Hospital, Memphis, TN USA; 2 Department of Immunology, St. Jude Children’s Research Hospital, Memphis, TN USA; 3 Department of Experimental Hematology, St. Jude Children’s Research Hospital, Memphis, TN USA

## Abstract

Sickle cell disease (SCD) is a major global health concern. Patients with SCD experience disproportionately greater morbidity and mortality in response to influenza infection than do others. Viral infection is one contributing factor for the development of Acute Chest Syndrome (ACS), a major cause of morbidity and mortality in SCD patients. We determined whether the heightened sensitivity to influenza infection could be reproduced in the two different SCD murine models to ascertain the underlying mechanisms of increased disease severity. In agreement with clinical observations, we found that both genetic and bone marrow-transplanted SCD mice had greater mortality in response to influenza infection than did wild-type animals. Despite similar initial viral titers and inflammatory responses between wild-type and SCD animals during infection, SCD mice continued to deteriorate and failed to resolve the infection, resulting in increased mortality. Histopathology of the lung tissues revealed extensive pulmonary edema and vascular damage following infection, a finding confirmed by heightened vascular permeability following virus challenge. These findings implicate the development of exacerbated pulmonary permeability following influenza challenge as the primary factor underlying heightened mortality. These studies highlight the need to focus on prevention and control strategies against influenza infection in the SCD population.

Sickle cell disease (SCD) is one of the most common inherited autosomal recessive disorders worldwide. In the United States alone, SCD affects about one in every 500 African-American infants. Patients with SCD have not only hematological complications but also pulmonary complications such as pulmonary hypertension. One particular pulmonary complication, acute chest syndrome (ACS), is a common complication of the sickling disorders and is a major cause of morbidity and mortality associated with SCD^1^,^2^. While numerous noninfectious causes of ACS have been described, infectious diseases, especially respiratory pathogens, are also considered to be a contributing factor in the development of ACS in these patients^3^,^4^,^5^. Influenza virus has long been known to cause disproportionate morbidity in those patients with SCD^6^. Highlighted by the 2009 influenza pandemic, SCD itself is considered to be an independent risk factor for increased risk of severe complications, death, or both from influenza infection^7^,^8^. Influenza-associated hospitalization rates have been estimated to be up to 56 times greater in children with SCD than in other children^7^,^9^ In addition, the risk of influenza infection is compounded by influenza vaccine coverage as low as 22% in the SCD population and a paucity of data on the effectiveness of antiviral therapy in this high-risk population^10^,^11^,^12^. Chronic transfusions used to treat SCD can also impair the immune response to influenza vaccines in this population^13^. Therefore, despite available interventions, influenza infection remains a serious health concern for patients with SCD.

While numerous clinical reports have confirmed the increased morbidity and mortality associated with influenza infection in SCD patients, to date, no laboratory models have investigated influenza infection in a murine model of SCD. The mechanisms underlying the exacerbated morbidity and mortality that patients with SCD undergo during influenza virus infection remain unresolved. Increased susceptibility has been hypothesized to result from SCD-associated immune defects; however, questions about the exact causes underlying the heightened susceptibility linger. Therefore, to begin to understand the dynamic interplay of influenza virus and those with SCD, we questioned whether a murine model of SCD would recapitulate the heightened sensitivity observed in the clinical setting.

In this study, we found that both genetic- and bone marrow transplant–induced SCD mice displayed heightened morbidity and mortality after influenza challenge, reflecting the clinical outcomes in SCD patients. Despite having similar viral loads at early time points of infection and minimal differences in inflammatory response to infection, SCD animals’ viral clearance ability was impaired compared to that of wild-type animals. At time points where wild type animals begin to resolve the infection, the SCD animals continue to deteriorate, with significantly enhanced pulmonary damage and edema characteristic of the acute injury associated with ACS^14^,^15^,^16^. While numerous other murine models of ACS have been created that have no infectious etiology^17^,^18^,^19^, these data implicate that development of ACS-like symptomology could be contributing to the enhanced mortality to influenza infection in SCD and provide the first animal model for influenza virus-induced ACS in SCD.

## Design and Methods

### Generation of SCD mice

All experiments and procedures were approved by the Animal Care and Use Committee at St. Jude Children’s Research Hospital and experiments were carried out in accordance with approved guidelines. Experiments were conducted with 8–12 week old BERK SCD mice^20^, heterozygous littermate controls (WT), and bone marrow–transplanted mice (SCD_BM_ and WT_BM_). Bone marrow–transplanted mice were generated as previously described^21^. Briefly, lethally irradiated 8-week-old female C57B/J6 mice (Jackson Labs, Bar Harbor, ME) were transplanted as described previously, receiving 2 × 10^6^ bone marrow cells from either BERK SCD mice or WT mice^21^. The sickle phenotype was confirmed by performing hemoglobin cellulose acetate electrophoresis of red cell lysates^22^. Complete blood counts were determined to ascertain the number of white blood cells, hematocrit and hemoglobin values, and red blood cell distribution.

### Influenza infection and tissue collection

WT and SCD mice were lightly anesthetized with isofluorane and intranasally inoculated with 10^2^ TCID_50_ units of influenza A/California/04/2009 (pdmH1N1) in 25 μL PBS. Mice were monitored daily for clinical signs of infection^23^ and weighed every 24 hours post-inoculation. At days 0 (uninfected), 3, 6, 10, and 14 post-infection, mice (n = 3/time point per experiment) were euthanized and whole lung, spleen, bronchoalveolar lavage fluid (BALF), and nasal washes were collected. Lung tissue was homogenized in 1 mL PBS for further analyses.

### Antiviral drug treatment

Oseltamivir (Roche) was prepared as described previously, and treatment (0, 20, or 50 mg/kg by oral gavage twice daily) was initiated starting at the day of infection and continued for 5 days^24^.

### Viral titer determination

Viral titers of lung homogenates, BALF, and nasal washes were determined by 50% tissue culture infectious dose (TCID_50_) on Madin-Darby canine kidney (MDCK) cells^25^. Briefly, MDCK cells were infected with 100 μL of 10-fold serial dilutions of sample and incubated at 37 °C for 72 hours. After incubation, viral titers were determined by using 0.5% turkey red blood cells to perform hemagglutination assays and analyzed by using the method of Reed and Munch^26^.

### Histological analysis

After euthanasia, mice (n = 3–5/time point per experiment) were perfused with 3% paraformaldehyde and lungs were excised and instilled with paraformaldehyde under constant pressure to expand the lungs to approximately physiological dimensions. For histopathology, lungs were collected at days 6 and 10 post-infection and immediately perfused with 10% formaldehyde and embedded in paraffin for hematoxylin and eosin (H&E) staining.

### Cytokine/chemokine measurement

Levels of cytokines and chemokines were measured by using the Milliplex MAP kits (Millipore) according to the manufacturer’s guidelines.

### Isolation of cells from spleen and BALF

Samples were processed into single-cell suspensions by mechanical agitation and strained through a 40-μm nylon filter. Then, cells were subjected to red blood cell lysis by using ACK lysis buffer for 5 min at room temperature, washed, counted, and stained for flow cytometry analysis.

### Flow Cytometry

BAL, mediastinal lymph node, and spleen were harvested from mice 7 days after intranasal inoculation with mouse-adapted A/California/04/2009 (H1N1). The organs were processed to produce single-cell suspensions. At least 1 × 10^6^ cells were Fc-blocked with anti-CD16/CD32 in FACS buffer (DPBS + 0.1% BSA, 0.02% NaN_3_). Samples were stained with allophycocyanin (APC)-anti-Cd11b, phycoerythrin (PE)/Cy5-anti-CD11c, Pacific Blue-anti-MHCII, APC/Cy7-anti-Gr1, fluorescein isothiocyanate (FITC)-anti-NK1.1-FITC, and PE-anti-CD80-PE antisera for 1 hour; washed; and fixed with FACS Buffer + 1% HCHO to detect innate immune cells. Adaptive immune cells were intracellularly stained for 1 hour with tetramers targeting the influenza D^b^NP_366_ and K^b^PB1_703_ epitopes (NP-PE and PB1-APC). After intracellular staining, adaptive cells were washed and exposed to FITC-anti-NK1.1, Brilliant Violet 785-anti-CD4, Pacific Blue-anti-CD8, peridinin-chlorophyll-protein complex (PerCP)/Cy5.5-anti-CD27, and APC/Cy7-anti-CD43 for 1 hour, then washed and fixed with FACS buffer + 1% HCHO. Samples were analyzed by using an LSRII flow cytometer running FACSDiva software (BD Biosciences), and data were analyzed by using FlowJo software (TreeStar). For innate cell gating, CD11b^+^CD11c^−^ cells were considered to be inflammatory monocytes; NK1.1^+^ cells were considered natural killer cells; and CD11b^+^, CD11c^−^, Gr1^+^, and MCHII^−^ cells were considered to be neutrophils.

### Permeability Assays

At day 6 post-inoculation, mice were administered 20 mg/kg Evans blue dye (Sigma) by retro-orbital injection. The dye was allowed to circulate for 2 hours before mice were euthanized: blood was collected for serum analyses, and the mice were perfused with 30 mL PBS. Lungs were harvested and incubated in formamide (Sigma) at 65 °C. After 48 hours, lungs were removed from formamide, dried at 60 °C for an additional hour, and then weighed. Evans blue dye coloration was measured in lung and serum by spectrophotometry at 620 nm. The lung permeability index was calculated as previously described by using the following formula: ([(lung absorbance/serum absorbance) × dilution factor]/dry lung weight [g])^27^. For measurement of liquid content of the lungs following infection, lungs were harvested at day 6 post challenge and the wet weights determined. Following three days at 65 °C lungs were re-weighted re-weighed and the ratio of wet: dry weights calculated.

### Blood Oxygen Measurements

Blood oxygen levels were measured on day −2 for baseline SpO_2_ using the MouseSTAT Jr. Pulse Oximeter for Mice & Rats (Kent Scientific) and again on day 6 post infection. Briefly, mice were anesthetized using 2.5% isoflurane and kept under anesthesia during monitoring. The hind paw of the mouse was placed in the paw sensor, with the pad directly over the red light. SpO_2_ levels were recorded for each mouse.

### Statistical analysis

Statistical analysis was conducted by using Prism (GraphPad) and JMP (SAS Corporation) software. Survival of murine groups was compared by log-rank testing. Differences in viral titers and cytokines were compared by Mann-Whitney testing. For all experiments, p < 0.05 was considered to be significant.

## Results

### Murine transplant sickle cell mice have increased morbidity and mortality following influenza virus infection

**Figure 1:**
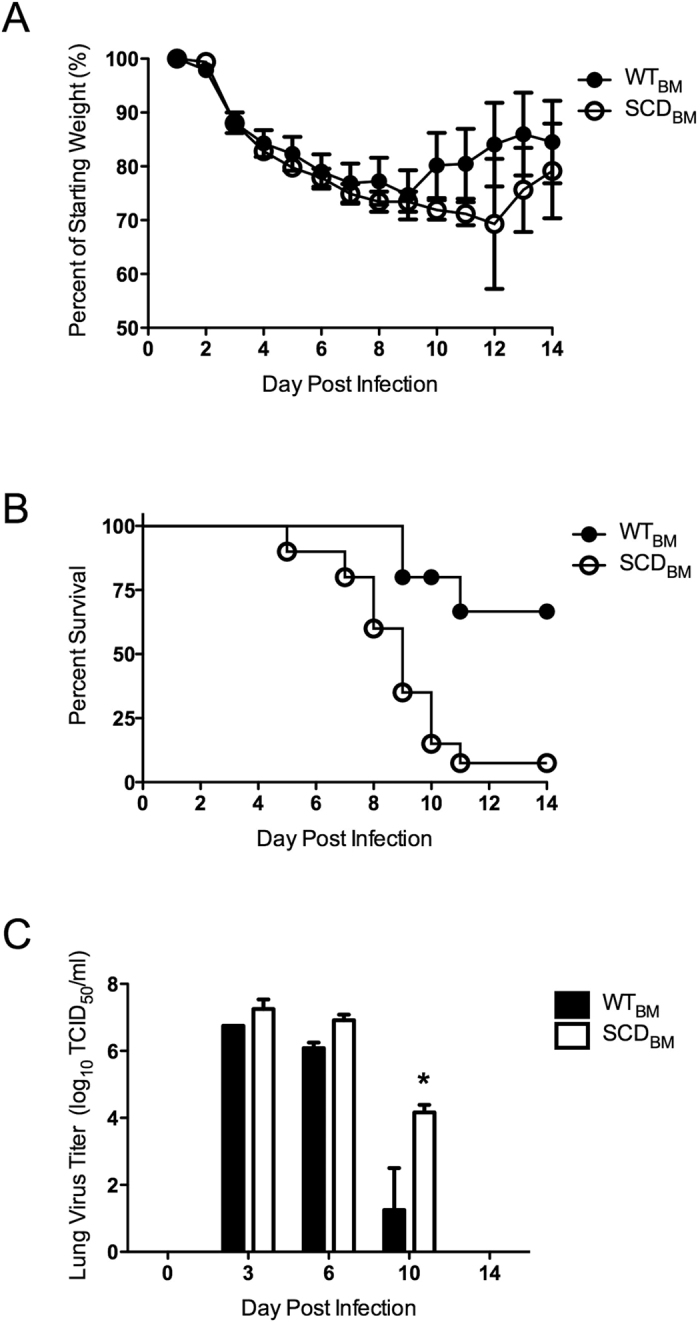
Heightened susceptibility in transplanted SCD mice during influenza infection. (**A**) Weight loss and (**B**) survival of transplanted WT_BM_ (n = 20, 15 survivors) and SCD_BM_ (n = 18, 2 survivors) mice after influenza inoculation (p =  < 0.0001). *p < 0.05 by log-rank test. (**C**) Lung viral titers (n = 3/group) after inoculation. *p < 0.05 by paired *t*-test. Data and error bars represent the mean and standard error for (**A** and **C**).

We first sought to determine whether the heightened mortality of influenza virus in patients with SCD could be recapitulated in the SCD transplant murine model. After confirmed engraftment, SCD_BM_ and WT_BM_ mice were challenged with the 2009 pandemic strain of influenza virus. SCD_BM_ mice did not display significantly greater weight loss than WT_BM_ controls did (Fig. 1A). However, SCD_BM_ mice had significantly increased mortality starting at day 5 post-inoculation, resulting in only 10% survival in SCD_BM_ mice by day 14 post-inoculation (Fig. 1B).

### Early influenza virus burden is not different between transplanted sickle cell and wild type hosts

One predictive measure of mortality during influenza infection is the viral burden. Viral titers of lung tissue from both groups had similar initial kinetics and overall magnitude at early stages of infection, being indistinguishable at days three and six post-inoculation (Fig. 1C). These results suggest that replication and overall viral burden were similar in magnitude and kinetics at these early phases of infection, prior to the time at which the WT_BM_ and SCD_BM_ animals began to diverge in terms of disease outcomes. Only at day ten post-challenge was a significant difference in lung viral titers observed, with the SCD_BM_ animals having viral loads significantly greater than those of the WT_BM_ animals, which had begun the process of viral clearance (Fig. 1C). These data indicate that the increased mortality observed clinically during influenza infection can be effectively modeled in the transplant murine model of SCD.

### Differences in influenza morbidity and mortality are similar between genetic and transplant models of sickle cell disease in mice

**Figure 2:**
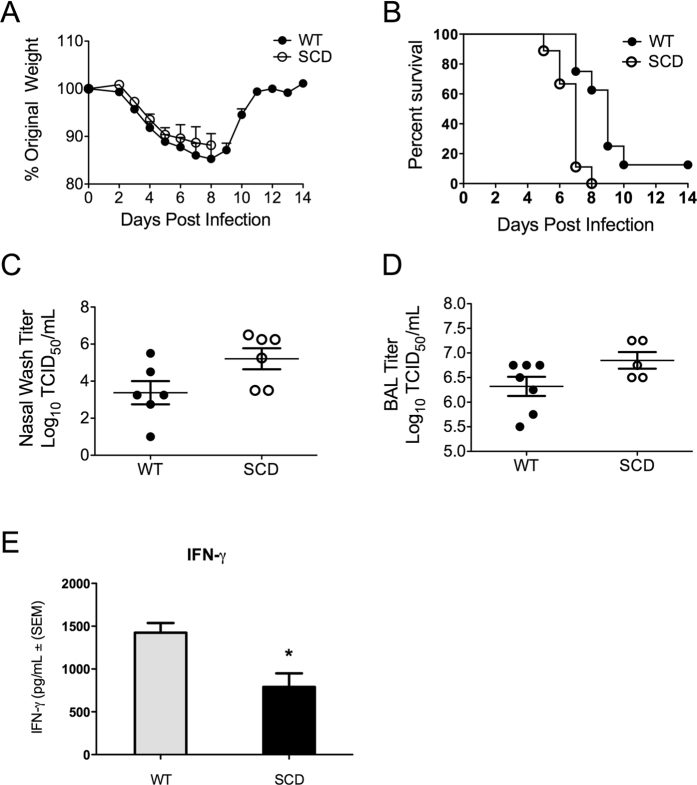
Susceptibility of BERK SCD mice to influenza. (**A**) Weight loss after influenza challenge in BERK SCD mice (SCD) and heterozygous littermate controls (WT). (**B**) Survival kinetics of mice in A after influenza inoculation (WT: 1 survivor, SCD: no survivors). *p < 0.05 by Log rank test. n = 10 mice per group (**C**,**D**) Viral titers recovered from nasal and lung lavages of mice at day 6 post- inoculation. (**E**) Levels of IFN-γ in the BALF of mice at day 6 post–inoculation. Each data point represents an individual mouse. *p < 0.05 by log rank test. Data and error bars represent the mean and standard error for (**A**,**C**,**D** and **E**).

We next sought to determine whether the heightened susceptibility to influenza infection observed in the transplant model could be recapitulated in BERK SCD mice and heterozygous littermate controls (WT). Due to the limitations of both bone marrow transplant and, in the case of the non-transplanted mice, a poorly defined mixed genetic background, use of both systems would be advantageous to query various aspects of infection susceptibility. Despite having no differences in weight loss (Fig. 2A), the BERK SCD mice had a mortality rate that was significantly greater than that of their heterozygous littermate controls (Fig. 2B), similar to the results of transplanted mice. WT_HET_ were more susceptible to influenza infection than were WT_BM_ transplant controls; however, this difference could be due to their genetic background being mixed and distinct from that of the highly influenza-resistant C57Bl/6 model^28^. At day 6 post-inoculation, the BERK SCD mice had significantly higher viral loads in the nasal wash (Fig. 2C) and equivalent titers in the BALF (Fig. 2D) though the SCD animals averaged greater loads in the lungs. These data indicate that heightened susceptibility to influenza can be effectively modeled in both the BERK and transplant murine models of SCD.

### SCD have only minimal differences in inflammatory cytokine expression following influenza virus infection compared to healthy controls

We next sought to determine what differences in cytokine levels during influenza infection might underlie the observed hyper-inflammatory state in SCD (Supplementary Figure 1). In contrast to the case during bacterial infection, similar TNFα and IL-6 levels were observed between the virus-infected WT and SCD animals. Both IL-1α and IL-1β levels in the SCD animals were elevated at baseline, similar to levels eventually reached by these cytokines in WT animals after virus infection. IL-10 and MIP-1β were also elevated in SCD animals after influenza infection. The levels of IFN-γ were also significantly decreased in the SCD animals following challenge (Fig. 2E). These data indicate that influenza infection in SCD is not associated with markedly greater levels of inflammatory cytokines.

### SCD mice have only minor differences in cellular inflammatory infiltrate compared to healthy controls

**Figure 3:**
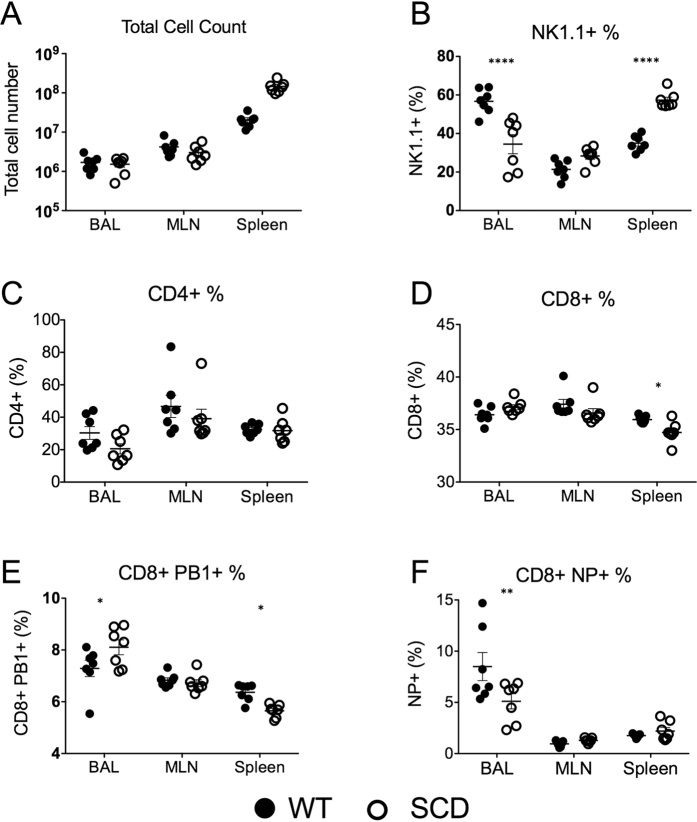
Immune cell profiles of BERK SCD mice and heterozygous littermate controls at day-7 post-influenza inoculation. Immune cells were stained and quantitated by flow cytometry analysis of cells collected from the lungs (BAL), lymph nodes (MLN), and spleen. *p < 0.05, **p < 0.01, ****p < 0.001 by 2-way ANOVA. Black circles are WT mice and white circles are SCD animals. Data and error bars represent the mean and standard error.

In addition to determining levels of inflammatory cytokines after infection, we also analyzed the cellular inflammatory infiltrate into the lungs in both the SCD BERK and WT control mice. At 6 days post-challenge, no significant differences were found in the total number of lymphocytes in the BALF, nor were significant differences observed in the total numbers of neutrophils, inflammatory monocytes, natural killer T cells, CD4^+^ T cells, or CD8^+^ T cells. The SCD mice had a significantly decreased proportion of NK T-cells in the BALF after challenge (Fig. 3B). There was also a slight, but significant decrease in the proportion of NP^+^ CD8^+^ T cells in the SCD animals (Fig. 3E). These data indicate that although most of the influenza-mediated inflammatory response in SCD animals was indistinguishable from that of heterozygote controls, significant differences in the ratios of T-cell populations, particularly NK cells, were observed. Overall, however, the inflammatory infiltrate of the SCD mice and heterozygous controls was markedly similar in terms of absolute numbers of cellular infiltrate.

### SCD mice have significantly increased pulmonary permeability following influenza virus infection

**Figure 4:**
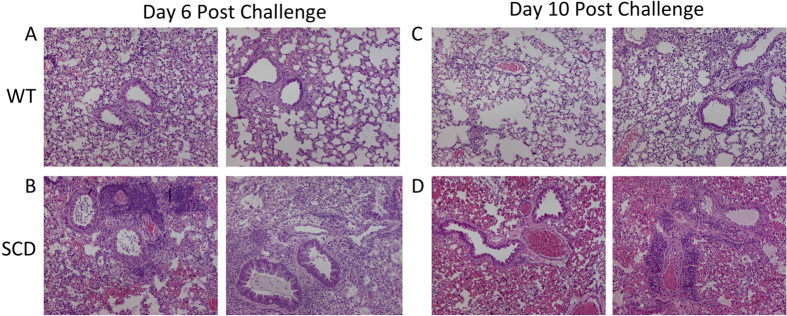
Representative images from hematoxylin and eosin staining of the lungs harvested from WT mice at days 6 and 10 post-inoculation (**A**,**C**) and SCD mice at days 6 and 10 post-inoculation (**B**,**D**).

Although the inflammatory response to virus challenge appeared to be largely operative, the SCD animals continued to deteriorate and had poorer disease outcomes. One of the major complications of virus infection in SCD patients is the development of ACS, which is characterized by pulmonary edema and plural effusions. Because heightened vascular permeability can lead to increased pulmonary edema, decreased lung function, and delayed wound repair, we examined whether increased pulmonary permeability was playing a significant role in SCD mortality during influenza challenge. To test this, we examined pulmonary damage subsequent to infection by H1N1 pandemic influenza virus in the context of SCD. At day six post-inoculation, WT animals had widespread areas of leukocyte infiltration into alveoli and interstitial spaces that was accompanied by extensive thickening or obliteration of alveolar walls (Fig. 4A). In contrast, the SCD animals had dense consolidation, with more intense intra-alveolar hemorrhage resulting in red hepatization of the tissue (Fig. 4B). By day ten after inoculation, the lungs of the WT_HET_ animals were resolving, demonstrating only mild pneumonitis with minimal perivascular leukocyte infiltration coupled with patchy, mild thickening of the alveolar walls (Fig. 4C). This finding was in stark contrast to that in the SCD animals, which had severe intra-alveolar hemorrhage (Fig. 4D). These data suggest influenza infection was causing pulmonary damage in a manner reminiscent of what is observed in SCD patients during ACS.

**Figure 5:**
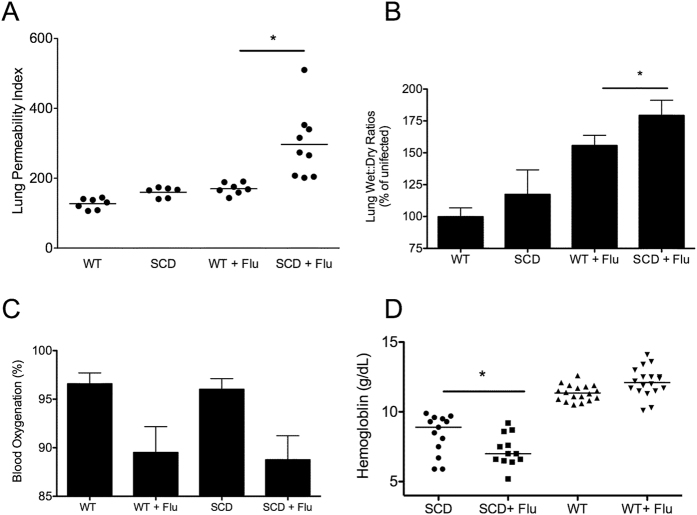
Pulmonary permeability and heightened anemia following influenza infection. Uninfected WT and SCD mice had similar levels of pulmonary permeability using Evans Blue assay (**A**) but SCD displayed greater permeability following challenge compared to infected Het controls (n = 6–9 per group). Wet-dry ratios following infection show a similar pattern whereby the SCD lungs had a significantly greater wet: dry ratio compared to heterozygous controls. All samples were normalized to uninfected WT control mice (wet: dry ratio = 3.13). Following challenge both WT and SCD mice displayed a significant reduction in blood oxygenation but no significant difference between het and SCD was observed following infection (**C**), n = 7–10 per group. Following influenza challenge SCD had a significant decrease in hemoglobin levels at day 6 post-challenge (**D**), n = 12–18 per group. For all figures *p < 0.05 by Mann-Whitney. Data and error bars represent the mean and standard error where applicable.

As an additional confirmation of this finding, the permeability of the lungs was assayed by using an Evans Blue dye assay both at baseline and after infection^27^,^29^. This accepted method of monitoring acute lung injury is based on the translocation of the dye into the lungs following intravenous injection allows for a measure of pulmonary permeability as the dye travels from the blood across the endothelium and through the epithelium into the alveolar space^29^. The WT and BERK SCD animals had similar permeability indexes at baseline, indicating an intact pulmonary architecture in the absence of infection. After infection, only modest increases in permeability (~50%) were observed in the heterozygous animals. In contrast, the SCD animals underwent a dramatic increase in pulmonary permeability (~200%) post-infection (Fig. 5A), indicating that an increased extent of vascular permeability is indeed occurring following influenza challenge in SCD. These data in conjunction with the histopathology suggested the SCD animals had heightened levels of pulmonary edema. This was confirmed via measurement of the wet/dry ratios of the lung weights following influenza challenge at day 6 post-challenge. These data confirmed that the SCD animals had significantly greater fluid accumulation in their lungs following influenza challenge at the peak of infection (Fig. 5B) compared to heterozygous control animals. Following infection both SCD and WT animals had similar decreases in blood oxygenation (Fig. 5C). In addition, SCD animals demonstrated a slight but significant decrease in hemoglobin levels compared to the levels in the same animals prior to infection (Fig. 5D) in concordance with observation in human SCD patients during ACS^30^. These data indicate that although the inflammation in SCD mice is only slightly altered at the early stages of infection compared to their state in healthy hosts, the SCD animals continue to deteriorate at later stages of infection, with continued destruction of pulmonary architecture was impaired, reminiscent of the clinical manifestations of ACS.

### Oseltamivir less effective at reducing mortality in SCD mice

**Figure 6:**
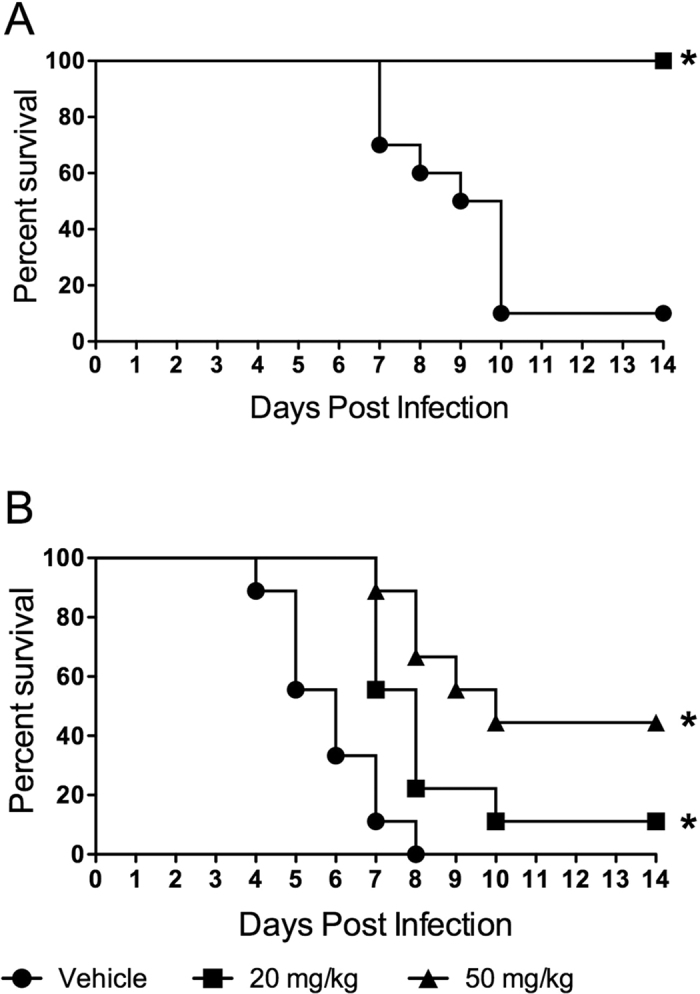
Effectiveness of antivirals in protecting SCD mice from influenza. Protective capacity of oseltamivir in WT (**A**) and SCD mice (**B**). Daily dosage of oseltamivir is indicated in figure legend. *p < 0.05 by Mantel log –rank test compared to vehicle controls. DPI = days post- infection. n = 10 mice per group. Vehicle = no survivors, 20 mg/kg = 1 survivor, 50 mg/kg = 4 survivors).

As influenza vaccine coverage in the SCD patient population can be significantly lower than the general population^10^,^12^,^13^, the efficacy of antiviral therapy is a critical question, particularly given the observation of enhanced edema mortality despite equivalent viral loads. This might indicate that antivirals may not be as effective in preventing mortality in SCD or require a higher dose than would be required in the general population to improve patient outcomes. Thus, we sought to utilize the SCD mouse model to ascertain the effectiveness of oseltamivir in preventing the mortality associated with influenza infection in the context of SCD. Treatment of WT mice with 20 mg/kg of oseltamivir resulted in a dramatic improvement of influenza-associated mortality in agreement with previously published reports (Fig. 6A)^31^. In the SCD animals, administration of oseltamivir significantly delayed mortality at both 20 mg/kg and 50 mg/kg treatment groups although overall mortality was only significantly improved at the 50 mg/kg dosage (Fig. 6B). Even at the higher dosage, protection from mortality was not as dramatic as that observed in wild type animals receiving the lower dose of antivirals. Hence, the period of time for effective administration of such antivirals to prevent mortality in SCD is likely distinct from the general population. These data underscore the importance of early intervention and vaccination to reduce morbidity and mortality associated with influenza infection in the context of SCD.

## Discussion

In developed countries, ACS is the second most common cause of hospitalization and premature death in patients with SCD. Infectious diseases, especially of the upper and lower respiratory tract, are thought to be a possible trigger of ACS in patients with SCD, with excessive inflammatory lung injury thought to be one cause of ACS development^1^. During the 2009 H1N1 pandemic, several epidemiological studies showed that influenza infection was significantly associated with development of ACS and that the risk of ACS from pH1N1 infection was almost 3-fold higher than that associated with previous seasonal influenza epidemics^7^,^32^. Although this enhanced susceptibility is well known to occur in this patient population, the specific mechanism underlying this observation is poorly understood.

Our study indicates that the hyper-susceptibility to influenza infection observed in SCD patients can be effectively recapitulated in two murine models of SCD. We observed increased lethality in response to infection with pandemic influenza. While mortality did not correlate with initial or maximal viral titers, the SCD mice displayed defects in viral clearance at later time points indicative of a defect in the immune response to influenza infection. Indeed, one potential mechanism for this finding is the altered ratios of NK T-cells and decreased levels of IFN-gamma in SCD mice following influenza challenge. In terms of total cellular infiltrate following infection, the SCD animals were markedly similar to their littermate controls, indicating the immune response to infection was largely intact at the peak of infection.

We also sought to determine whether other factors might explain the increased mortality observed in the SCD animals. The exacerbation of pulmonary permeability observed in SCD mice after influenza infection indicate that this is a major contributing factor to the heightened morbidity and mortality. SCD animals displayed a high degree of fluid leakage into the lungs following influenza challenge, which was observed by both histopathology and direct measurements. This constellation of findings is consistent with diffuse alveolar damage suggestive of ACS. It is well established that lung edema and vascular damage are major contributing factors to influenza pathogenesis and both appear to be significantly amplified in SCD^33^. Further in-depth studies are warranted on pulmonary physiology in these animals to continue to understand influenza infection-associated changes in the SCD lung environment. In addition, our findings show that patients with SCD may require increased dosages of antiviral therapies for effective treatment. Murine modeling of the immune responses of SCD mice to vaccination also indicate an altered response to vaccination^34^. Coupled with the low vaccination in this population, early intervention with antivirals and increased vaccine usage are crucial in preventing and treating influenza in SCD patients. Overall, these murine SCD models of influenza-induced morbidity and mortality will be invaluable for studying prophylactic and therapeutic strategies in the high-risk SCD population.

## Additional Information

**How to cite this article:** Karlsson, E. A. *et al*. Vascular Permeability Drives Susceptibility to Influenza Infection in a Murine Model of Sickle Cell Disease. *Sci. Rep.* **7**, 43308; doi: 10.1038/srep43308 (2017).

**Publisher's note:** Springer Nature remains neutral with regard to jurisdictional claims in published maps and institutional affiliations.
